# Supplementary material for: Comparing self-reported and O*NET-based assessments of job control as predictors of self-rated health for non-Hispanic whites and racial/ethnic minorities
Source: PLoS One. 2020 Aug 6;15(8):e0237026. doi: 10.1371/journal.pone.0237026 (PMC7410273; doi:10.1371/journal.pone.0237026)
Supplement: S1 Table — (DOCX) [file pone.0237026.s001.docx]

**S1 Table. Frequencies of responses to Hispanic ethnicity and race questions by gender (n=7407)**

|  | Hispanic ethnicity | | |
| --- | --- | --- | --- |
| Race | Yes | No | missing |
|  | Men (n=3,569) | | |
| White | 199 | 2,562 | 1 |
| Black | 10 | 418 | 1 |
| Other | 225 | 151 | 2 |
|  | Women (n=3,838) | | |
| White | 228 | 2,579 | 0 |
| Black | 23 | 671 | 1 |
| Other | 181 | 151 | 4 |
