# Supplementary material for: Comparing self-reported and O*NET-based assessments of job control as predictors of self-rated health for non-Hispanic whites and racial/ethnic minorities
Source: PLoS One. 2020 Aug 6;15(8):e0237026. doi: 10.1371/journal.pone.0237026 (PMC7410273; doi:10.1371/journal.pone.0237026)
Supplement: S2 Table — (DOCX) [file pone.0237026.s002.docx]

**S2 Table. Psychometric properties and descriptive statistics of O*NET and self-reported measures of job control by gender and race**

|  |  | Self-report | | | | O*NET | |
| --- | --- | --- | --- | --- | --- | --- | --- |
| Respondents | *n* | Cronbach’s alpha | ICC1 | Mean (SD) | Range | Mean (SD) | Range |
| All respondents | 7041 | .70 | .18[.16, .22] | 3.24 (0.50) | 1-4 | 71.05 (10.30) | 37.06-91.82 |
| Without jobs <10 respondents | 5948 | .70 | .18[.16, .23] | -- | -- | -- | -- |
|  |  |  |  |  |  |  |  |
| *White men* | *2459* | *.72* | *.25 [.22, .31]* | 3.29 (0.50) | 1-4 | 71.50 (9.95) | 37.06-91.82 |
| *Non-white men* | *937* | *.68* | *.19 [.16, .29]* | 3.17 (0.49) | 1.17-4 | 70.71 (10.07) | 42.46-91.82 |
|  |  |  |  |  |  |  |  |
| *White women* | *2449* | *.71* | *.20 [.18, .27]* | 3.27 (0.49) | 1-4 | 71.97 (10.36) | 37.06 – 90.71 |
| *Non-white women* | *1196* | *.66* | *.16 [.15, .27]* | 3.15 (0.51) | 1-4 | 71.16(10.45) | 42.46 – 91.82 |

*Note*. All descriptive statistics are weighted, except for the range and *n*.
