# Supplementary material for: Comparing self-reported and O*NET-based assessments of job control as predictors of self-rated health for non-Hispanic whites and racial/ethnic minorities
Source: PLoS One. 2020 Aug 6;15(8):e0237026. doi: 10.1371/journal.pone.0237026 (PMC7410273; doi:10.1371/journal.pone.0237026)
Supplement: S3 Table — (DOCX) [file pone.0237026.s003.docx]

**S3 Table. The association between self-rated health (poor/fair) and job control by gender.**

|  | Model 1 | | Model 2 | |
| --- | --- | --- | --- | --- |
| Independent variable | OR (95%CI) | OR (95%CI) | OR (95%CI) | OR (95%CI) |
|  | Men (*n* = 3,396, *N* = 379) | | | |
| Job control |  |  |  |  |
| O*NET | 0.70 (0.60 – 0.81) |  | 0.64 (0.54 – 0.75) |  |
| Self-report (reduced) |  | 0.71 (0.65 – 0.78) |  | 0.71 (0.63 – 0.80) |
| Racial/ethnic minority | 1.44 (1.15 – 1.81) | 1.53 (1.25 – 1.89) | 1.58 (1.25 – 2.01) | 1.53 (1.23 – 1.90) |
| Minority x Job control |  |  |  |  |
| O*NET |  |  | 1.32 (1.05 – 1.67) |  |
| Self-report (reduced) |  |  |  | 0.99 (0.81 – 1.20) |
|  | Women (*n* = 3,645, *N* = 337) | | | |
| Job control |  |  |  |  |
| O*NET | 0.71 (0.63 – 0.80) |  | 0.64 (0.55 – 0.74) |  |
| Self-report (reduced) |  | 0.77 (0.71 – 0.85) |  | 0.77 (0.69 – 0.87) |
| Racial/ethnic minority | 1.62 (1.31 – 2.00) | 1.57 (1.29 – 1.91) | 1.78 (1.42 – 2.22) | 1.57 (1.28 – 1.91) |
| Minority x Job control |  |  |  |  |
| O*NET |  |  | 1.29 (1.05 – 1.59) |  |
| Self-report (reduced) |  |  |  | 1.00 (0.83 – 1.20) |

*Notes*. Age and GSS survey year are controlled for in all models. The self-report job control measure used only 3 items.
