# Supplementary material for: Comparing self-reported and O*NET-based assessments of job control as predictors of self-rated health for non-Hispanic whites and racial/ethnic minorities
Source: PLoS One. 2020 Aug 6;15(8):e0237026. doi: 10.1371/journal.pone.0237026 (PMC7410273; doi:10.1371/journal.pone.0237026)
Supplement: S4 Table — (DOCX) [file pone.0237026.s004.docx]

**S4 Table. Available O*NET and QWL items that address job demands**

| O*NET | | Self-report (QWL) | |
| --- | --- | --- | --- |
| Item [element ID] | Response options | Item | Response options |
| ***Time*** |  |  |  |
| Reaction time: the ability to quickly respond (with the hand, finger, or foot) to a signal (sound, light, picture) when it appears. [1.A.2.c.1]  Perceptual speed: The ability to quickly and accurately compare similarities and differences among sets of letters, numbers, objects, pictures, or patterns. [1.A.1.e.3]  Response orientation: The ability to choose quickly between “two or more movements” in response to “two or more different signals” (lights, sounds, pictures). [1.A.2.b.3] | *How important*  1= Not important^1^  2= Somewhat important  3= Important  4= Very important  5= Extremely important  *What levels is needed*: Low (1) to High (7)^2^ | My job requires that I work very fast. | 1=Very true  2= Somewhat true  3= Not too true  4= Not at all true |
| How important to your current job is keeping a page set by machinery or equipment? [4.C.3.d.4] | 1= Not important at all  2= Fairly important  3= Important  4= Very important  5= Extremely important |  |  |
|  |  | I have enough time to get the job done. | 1=Very true  2= Somewhat true  3= Not too true  4= Not at all true |
| ***Workload*** |  |  |  |
|  |  | I have too much work to do everything well. | 1=Very true  2= Somewhat true  3= Not too true  4= Not at all true |
|  |  | How often are there not enough people or staff to get all the work done. | 1= Often  2= Sometimes  3= Rarely  4= Never |
