# Supplementary material for: Comparing self-reported and O*NET-based assessments of job control as predictors of self-rated health for non-Hispanic whites and racial/ethnic minorities
Source: PLoS One. 2020 Aug 6;15(8):e0237026. doi: 10.1371/journal.pone.0237026 (PMC7410273; doi:10.1371/journal.pone.0237026)
Supplement: S5 Table — (DOCX) [file pone.0237026.s005.docx]

**S5 Table. Correlation coefficients between O*NET and self-report job demands measures**

|  | Individual level  (Pearson’s *r*) | Cross level  (*β*) |
| --- | --- | --- |
| All respondents | .00 | .01 |
|  |  |  |
| Men |  |  |
| White men | .00 | .03 |
| Non-white men | -.08* | -.06 |
| Women |  |  |
| White women | .05** | .00 |
| Non-white women | -.01 | -.03 |

*Notes.*  All coefficients are significant at. *β* = regression coefficient from a mixed linear model with self-reported job control as the dependent variable and O*NET measure as the independent variable with occupation as the random effect.

* *p*<.05, ***p*<.01
