# Supplementary material for: Comparing self-reported and O*NET-based assessments of job control as predictors of self-rated health for non-Hispanic whites and racial/ethnic minorities
Source: PLoS One. 2020 Aug 6;15(8):e0237026. doi: 10.1371/journal.pone.0237026 (PMC7410273; doi:10.1371/journal.pone.0237026)
Supplement: S6 Table — (DOCX) [file pone.0237026.s006.docx]

**S6 Table. The association between self-rated health (poor/fair) and job control by racial/ethnic job composition: Workers in less white-dominated jobs^a^ (n=2,266, N = 100).**

|  | Model 1 | | Model 2 | |
| --- | --- | --- | --- | --- |
| Independent variable | OR (95%CI) | OR (95%CI) | OR (95%CI) | OR (95%CI) |
| Job control |  |  |  |  |
| O*NET | 0.80 (0.69 – 0.92) |  | 0.73 (0.61 – 0.88) |  |
| Self-report |  | 0.75 (0.68 – 0.84) |  | 0.77 (0.67 – 0.87) |
| Racial/ethnic minority | 1.24 (0.99 – 1.56) | 1.23 (1.00 – 1.53) | 1.52 (1.09 – 2.12) | 1.23 (0.99 – 1.52) |
| Minority x Job control |  |  |  |  |
| O*NET |  |  | 1.24 (0.95 – 1.61) |  |
| Self-report |  |  |  | 0.97 (0.78 – 1.19) |

*Notes*. Age and GSS survey year are controlled for in all models. Controlling for gender did not change the results substantially. ^a^ jobs with < 65.7 % non-Hispanic whites (the bottom tertile of the %white on the job).
