# Supplementary material for: Comparing self-reported and O*NET-based assessments of job control as predictors of self-rated health for non-Hispanic whites and racial/ethnic minorities
Source: PLoS One. 2020 Aug 6;15(8):e0237026. doi: 10.1371/journal.pone.0237026 (PMC7410273; doi:10.1371/journal.pone.0237026)
Supplement: S1 File — (DOCX) [file pone.0237026.s007.docx]

Data Access Information

The GSS data are publicly available from the General Social Survey Data Explorer: <https://gssdataexplorer.norc.org/variables/vfilter>. Interested researchers can create a user profile and select the desired data to download. For this study, we downloaded the following variables:

DV: HEALTH1

IVs: LEARNNEW

MYSKILLS

OPDEVEL

WORKDIFF

WKDECIDE

WKFREEDM

Meta-data: ID_

WTSSALL

YEAR

Moderators: SEX

RACE

HISPANIC

ETHNIC (as backup if HISPANIC was missing)

Control variables: AGE

YEAR

Inclusion criterion: WRKSTAT

Connector: OCC10

Discontinued QWL-items: LOTOFSAY

SETTHNGS

The O*NET database is publicly available from the O*NET Resource Center: <https://www.onetcenter.org/database.html>. We used items 4.C.3.b.8, 4.C.3.b.2, and 4.A.2.b.3. Because GSS provides the Census 2010 Occupation Codes but O*NET is based on the Standard Occupation Code (SOC), a crosswalk is necessary. The modified O*NET database (version 24) with the Census 2010 Occupation codes is available from NIOSH. Please contact Dr. Leslie MacDonald ([lmacdonald@cdc.gov](mailto:lmacdonald@cdc.gov)).
